# From design to efficiency: cobalt-based MOFs for efficient and stable electrocatalysis in hydrogen and oxygen evolution reactions†

**DOI:** 10.1039/d5ra00286a

**Published:** 2025-03-18

**Authors:** Junaid Khan, Anique Ahmed, Abdullah A. Al-Kahtani

**Affiliations:** a Department of Physics, Government Postgraduate Collage No. 1 Abbottabad Khyber Pakhtunkhwa Pakistan junaidkhan.nanotech@gmail.com; b Department of Higher Education Achieves and Libraries Government of Khyber Pakhtunkhwa Pakistan; c Faculty of Engineering Sciences, GIK Institute of Engineering Sciences and Technology Topi 23640 Khyber Pakhtunkhwa Pakistan; d Department of Chemical and Bilogical Engineering, Gachon University 1342 Seongnam-daero Seongnam 13120 Republic of Korea; e Chemistry Department, Collage of Science, King Saud University P. O. Box 2455 Riyadh 22451 Saudi Arabia

## Abstract

The pursuit of clean and renewable energy sources demands efficient technologies for hydrogen production, with water splitting emerging as a promising route. This study explores the use of Cobalt-based Metal–Organic Frameworks (Co-MOFs) as electrocatalysts for both the hydrogen evolution reaction (HER) and oxygen evolution reaction (OER). Two distinct Co-MOFs, synthesized with the organic linkers 5-nitroisophthalic acid (X1) and pyridine-2,6-dicarboxylic acid (X2), were designed and evaluated for their electrocatalytic performance. X1 exhibited suboptimal morphology and a low specific surface area, resulting in lower catalytical activity and restricting its suitability for long-term applications. In contrast, X2 exhibited exceptional catalytic efficiency with remarkably low overpotentials for both HER (151.7 mV) and OER (180 mV), alongside superior long-term stability. The enhanced electrocatalytic performance of X2 is attributed to its optimized morphology, superior metal-active site distribution, and robust structural integrity, making it an ideal candidate for large-scale water splitting. This work paves the way for the development of high-performance MOF-based electrocatalysts, offering insights for advancing hydrogen generation technologies.

## Abstract 2

This study systematically explores how variations in linker chemistry affect metal-active site distribution, stability, and reaction kinetics, providing a deeper understanding of structure/morphological-performance relationships.

## Graphical abstract

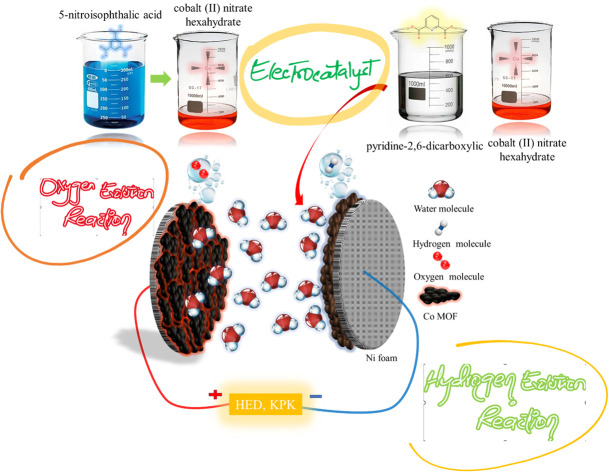

## 1. Introduction

The escalating demand for renewable energy solutions has brought hydrogen generation *via* water splitting to the forefront of energy research, fueled by the urgent need to transition from fossil fuels to sustainable alternatives.^1^ Storing renewable energy in the form of hydrogen is particularly advantageous because hydrogen, as a fuel, can seamlessly replace existing technologies with minimal modifications, offering a more practical solution compared to other alternatives.^2^ Water splitting, an electrochemical process, holds promise for clean hydrogen production, a cornerstone for future energy systems.^3^ The quest for efficient electrocatalysts to drive the oxygen evolution reaction (OER) and hydrogen evolution reaction (HER) has become critical to unlocking the potential of this technology.^4^ Conventional materials like IrO_2_ and RuO_2_ are widely used for OER due to their excellent catalytic activity and stability.^5^ However, their high cost and scarcity significantly limit large-scale applications.^6^ Similarly, for HER, platinum-based materials are considered state-of-the-art catalysts but suffer from similar economic and resource-based constraints.^7^ Various materials, including oxides, phosphates, sulfides, nitrides, carbon-based materials, and conducting polymers, have been extensively studied for water splitting.^8^ Metal oxides like Co_3_O_4_, NiO, and MnO_2_ are stable and catalytically active but underperform in HER.^9^ Phosphates are stable, cost-effective, and perform well in OER but are less efficient in HER and may degrade in basic conditions.^10^ Sulfides, particularly MoS_2_, excel in HER due to high surface area and efficient electron transfer, though stability and conductivity issues often require modifications.^11^ Carbon-based materials like graphene and CNTs offer excellent conductivity and large surface areas but need doping or functionalization to become catalytically active, and stability can be a concern under harsh conditions.^12^ Perovskite materials are tunable and catalytically efficient but expensive and prone to structural instability at high temperatures, limiting large-scale applications.^13^

These challenges underscore the need for alternative materials, which can provide a cost-effective and sustainable solution. Metal–Organic Frameworks (MOFs) have emerged as a compelling class of materials for catalytic applications, owing to their modular nature and structural versatility.^14^ MOFs, composed of metal nodes and organic linkers, exhibit characteristics such as tunable porosity, high surface area, and chemical adaptability, making them ideal candidates for tailoring catalytic properties.^15^ In water splitting reactions, the efficiency of a catalyst is largely determined by the overpotential, which is the additional voltage required beyond the thermodynamic equilibrium potential to drive the reaction. MOFs, while promising as catalysts for hydrogen evolution reaction (HER) and oxygen evolution reaction (OER), face significant challenges due to relatively high overpotentials.^16^ In HER, MOFs typically exhibit low intrinsic catalytic activity, with suboptimal hydrogen binding at their metal centers, leading to inefficient proton–electron coupling and higher overpotentials compared to more efficient catalysts like platinum.^17^ For OER, MOFs suffer from poor electrocatalytic properties, particularly for the O–O bond formation, which is a key step in the OER mechanism. The metal centers in MOFs often lack the necessary reactivity for efficient oxygen evolution, resulting in slower reaction kinetics and higher overpotentials than catalysts like iridium or ruthenium oxides.^18^ These high overpotentials hinder the catalytic efficiency of MOFs in water splitting applications. Despite their high surface areas, the limited conductivity of MOFs hinders catalytic efficiency, even when combined with conductive materials like carbon nanotubes or graphene, which adds complexity without fully resolving the issue.^16^ Additionally, MOFs are prone to structural degradation under harsh electrolytic conditions, with their weak metal–ligand bonds being susceptible to hydrolysis, particularly in the oxidative environments of OER. This instability affects both acidic and alkaline conditions, leading to framework collapse and loss of catalytic activity over time. A further limitation is the restricted number of active sites, as the metal nodes in MOFs are often not fully exposed or accessible, particularly in OER, where efficient O–O bond formation is critical.^19^ Slow reaction kinetics also impede their performance, with poor proton interactions in HER and suboptimal bond-breaking efficiency in OER resulting in reduced activity. Strategies like doping and defect engineering show promise but remain under development. Moreover, MOFs may exhibit high initial catalytic activity, long-term operation often results in diminished performance due to structural instability, limiting their potential for practical, large-scale water splitting.^20^

To address the challenges faced by MOFs in water splitting, strategic optimization of their structural and chemical properties is crucial. Enhancing the catalytic efficiency requires careful selection of metal nodes and organic linkers to reduce overpotentials and improve the stability of MOFs under harsh electrolytic conditions. For HER, optimizing the hydrogen binding energy at the metal centers can improve proton–electron coupling, while for OER, enhancing the reactivity of metal nodes can facilitate efficient O–O bond formation. This can be achieved by tailoring the coordination environment and electronic properties of the active sites through the use of suitable linkers and structural modifications.^21^ Cobalt-based Metal–Organic Frameworks (MOFs) are excellent candidates for water splitting due to their high surface area, tunable porosity, and active sites provided by cobalt centers.^22^ The intrinsic catalytic activity of cobalt enables efficient participation in both the oxygen evolution reaction (OER) and hydrogen evolution reaction (HER).^23^ Incorporating linkers with unique electronic properties, such as 5-nitroisophthalic acid and pyridine-2,6-dicarboxylic acid, can significantly enhance the catalytic performance of MOFs.^24^ These linkers can improve charge transfer within the framework and provide a robust architecture that minimizes structural degradation. The electron-withdrawing nature of 5-nitroisophthalic acid can enhance the catalytic reactivity of metal centers, while pyridine-2,6-dicarboxylic acid, with its chelating ability, can stabilize metal nodes and increase the number of accessible active sites.^25,26^ These linkers also allow for the synthesis of MOFs with tailored morphologies, such as thin nanosheets or hierarchical structures, which can further expose active sites and reduce diffusion limitations. Tailoring the surface morphology of MOFs offers a promising approach to addressing conductivity limitations and enhancing catalytic efficiency.^27^ Morphologies with higher surface areas and better accessibility to active sites facilitate faster charge transport and reduce diffusion limitations, thereby improving reaction kinetics for both HER and OER. Morphological modifications, coupled with robust linker and metal node selection, can mitigate slow reaction kinetics and address the structural degradation, achieve lower overpotentials, improved durability, and higher catalytic efficiency, paving the way for their practical application in large-scale water splitting.

In this study, cobalt-based MOFs (Co-MOFs) are synthesized by facile hydrothermal approach. Organic linkers, nitrous acid and 1,4-benzene dicarboxylic acid, are targeted to investigate their influence on the structural and catalytic properties of the MOF. Their surface morphologies, elemental analysis, surface area, crystal aspects specific surface area, stability, and catalytic efficiency were inspected. The outcomes of this work are expected to provide valuable insights into the design of MOF-based electrocatalysts, paving the way for innovative approaches to hydrogen generation *via* water splitting. By addressing the challenges of morphology control and catalytic efficiency, this study highlights the transformative potential of MOFs in achieving clean energy solutions.

## 2. Materials and methods

**Scheme 1:**
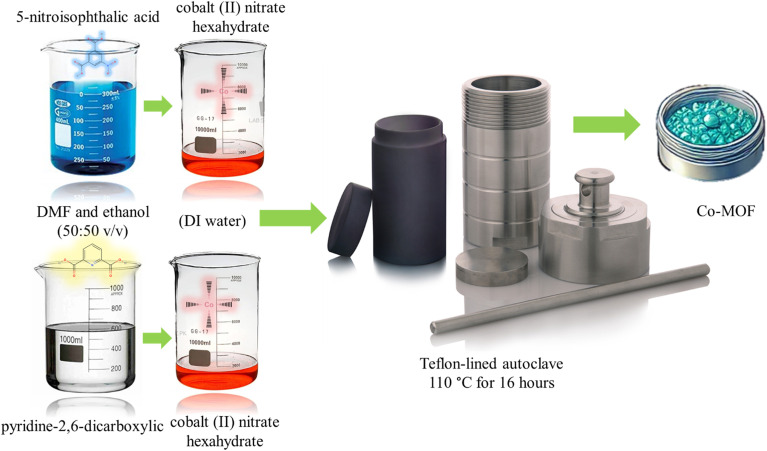
Schematic illustration of hydrothermal synthesis process adopted.

All the utilized materials were obtained from Sigma-Aldrich (analytical grade). Platinum wire and RHE are utilized as counter electrode and reference electrode. To synthesize the Co-MOF with 5-nitroisophthalic acid as the linker, 0.12 mol of 5-nitroisophthalic acid was dissolved in 20 ml of a solvent mixture comprising *N*,*N*-dimethylformamide (DMF) and ethanol (50 : 50 v/v) under constant stirring for 40 minutes. Simultaneously, 0.12 mol of cobalt(ii) nitrate hexahydrate was dissolved in 10 ml of DI water with stirring for 20 minutes. The two solutions were then combined dropwise under stirring and allowed to mix for an additional 3 hours at room temperature. The resulting mixture was transferred to a Teflon-lined autoclave and subjected to hydrothermal treatment at 110 °C for 16 hours. After cooling to room temperature, the obtained solid product was separated *via* centrifugation, washed multiple times with ethanol and DI water, and dried in an oven at 90 °C for 8 hours. The material was signified as X1. Similar synthesis approach was adopted but this time 0.12 mol of pyridine-2,6-dicarboxylic acid was utilized as a linker. The synthesized material was signified as X2. The synthesis process is schematically represented in Scheme 1.

X1 and X2 slurry was formulated by combining 80 wt% active material, 10 wt% PVDF and 10 wt% AB in NMP solvent. The mixture underwent continuous stirring for 8 hours to achieve homogeneity before being uniformly applied onto a clean 1 × 1 cm^2^ nickel foam. After deposition, the electrode was dried at 70 °C for 9 hours prior to analysis.

## 3. Results and discussion

### 3.1. Structural and morphological aspects

**Fig. 1:**
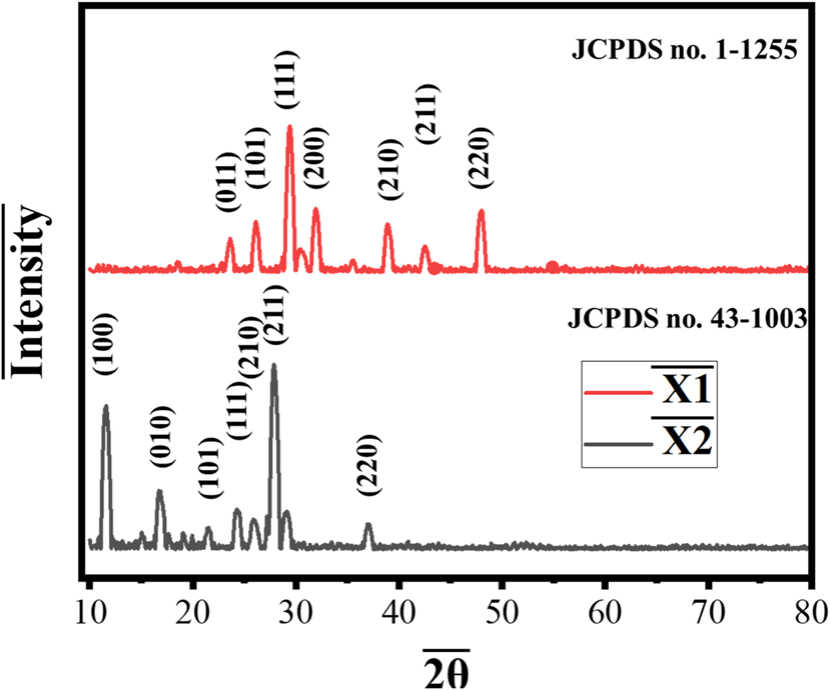
XRD characterization of the synthesized Co-MOFs, X1 and X2.

The XRD characterization of the synthesized Co-MOFs, X1 and X2, reveals key structural insights relevant to their application as water-splitting catalysts (Fig. 1). X1, synthesized using 5-nitroisophthalic acid as the organic linker, exhibited diffraction peaks at 2*θ* values of 23.5°, 26.1°, 29.3°, 31.9°, 39.0°, 42.6°, and 47.9°. These peaks indexed to the planes (011), (101), (111), (200), (210), (211), and (220). The sharp peaks at 26.1°, 29.3°, 31.9°, and 47.9° shows good correspondence with JCPDS no. 1-1255, indicates high crystallinity and preferential growth along specific orientations.^28^ This pronounced crystallinity is attributed to the strong coordination between cobalt ions and the nitro-functionalized linker, which promotes a highly ordered framework.^29^ Based on the peak distribution and comparison with related structures, X1 likely adopts a triclinic crystal system.^30^ Similarly, X2, synthesized using pyridine-2,6-dicarboxylic acid as the organic linker, displayed diffraction peaks at 2*θ* values of 11.5°, 16.6°, 21.3°, 24.2°, 27.9°, 29.2°, and 36.9°. The peaks are indexed to the planes (100), (010), (101), (111), (210), (211), and (220). Additionally, the pattern shows strong correspondence with JCPDS no. 43-1003.^31^ The shift of diffraction peaks toward low angle values indicates an expansion of the unit cell, which can be attributed to the influence of the pyridine-2,6-dicarboxylic acid linker.^32–34^ Compared to 5-nitroisophthalic acid in X1, the pyridine-based linker in X2 has a different steric and electronic effect, which alter the coordination environment around the cobalt centers.^35^ The sharp reflections again highlight the high crystallinity and structural integrity of X2. The aromatic pyridine groups in the linker promote directional growth, resulting in a uniform framework that enhances the material's catalytic properties.^36^ The crystalline nature of both X1 and X2, as evidenced by the sharpness and intensity of the XRD peaks, is crucial for their catalytic performance in water splitting.^37^ The ordered arrangement of cobalt active centers facilitates efficient charge transfer and minimizes resistance during the catalytic reaction.^38^ The preferential orientation of planes such as (101), (111), and (200) is associated with enhanced electronic conductivity and increased active site exposure, both critical for OER.^39^

Fig. S1(a) and (b)† present the FTIR spectra of the synthesized Co-MOFs, X1 and X2, respectively, confirming the successful incorporation of their respective organic linkers. In Fig. S1(a),† the characteristic stretching vibrations of the nitro (-NO_2_) functional group from 5-nitroisophthalic acid are observed in the region of 1500–1350 cm^−1^, along with a strong C=O stretching band around 1600 cm^−1^, indicating the presence of coordinated carboxylate groups.^40^ The broad peak around 3400 cm^−1^ corresponds to O–H stretching vibrations, suggesting the presence of surface hydroxyl groups or adsorbed moisture. Notably, the asymmetric and symmetric stretching vibrations of carboxylate groups at approximately 1600 cm^−1^ and 1380 cm^−1^ confirm the coordination of the organic linker with Co metal ions.^41^ Similarly, Fig. S1(b)† displays the FTIR spectrum of X2, synthesized using pyridine-2,6-dicarboxylic acid, where the characteristic peaks associated with pyridine ring vibrations appear around 1400–1600 cm^−1^. The peaks at ∼1620 cm^−1^ and ∼1380 cm^−1^ correspond to the asymmetric and symmetric stretching of the carboxylate groups, respectively, confirming their coordination with the Co metal centers.^42^ Additionally, the presence of pyridine-related stretching bands in this region further validates the incorporation of the organic linker within the Co-MOF structure. The observed spectral differences between X1 and X2 highlight the structural variations induced by the distinct linkers, reinforcing the successful synthesis and coordination of the organic linkers with Co metal ions.

Fig. S2† displays the nitrogen adsorption–desorption isotherm of X1 and X2 recorded at 77 K. The isotherm demonstrates a distinctive type I profile, characteristic of porous materials. Based on BET analysis, the surface area is determined to be 1690 and 2210 m^2^ g^−1^. Additionally, pore size distribution analysis verifies the existence of mesopores, with pore volumes of 0.9 and 1.1 cm^3^ g^−1^ for X1 and X2, respectively, affirming the better aspects of X2.

**Fig. 2:**
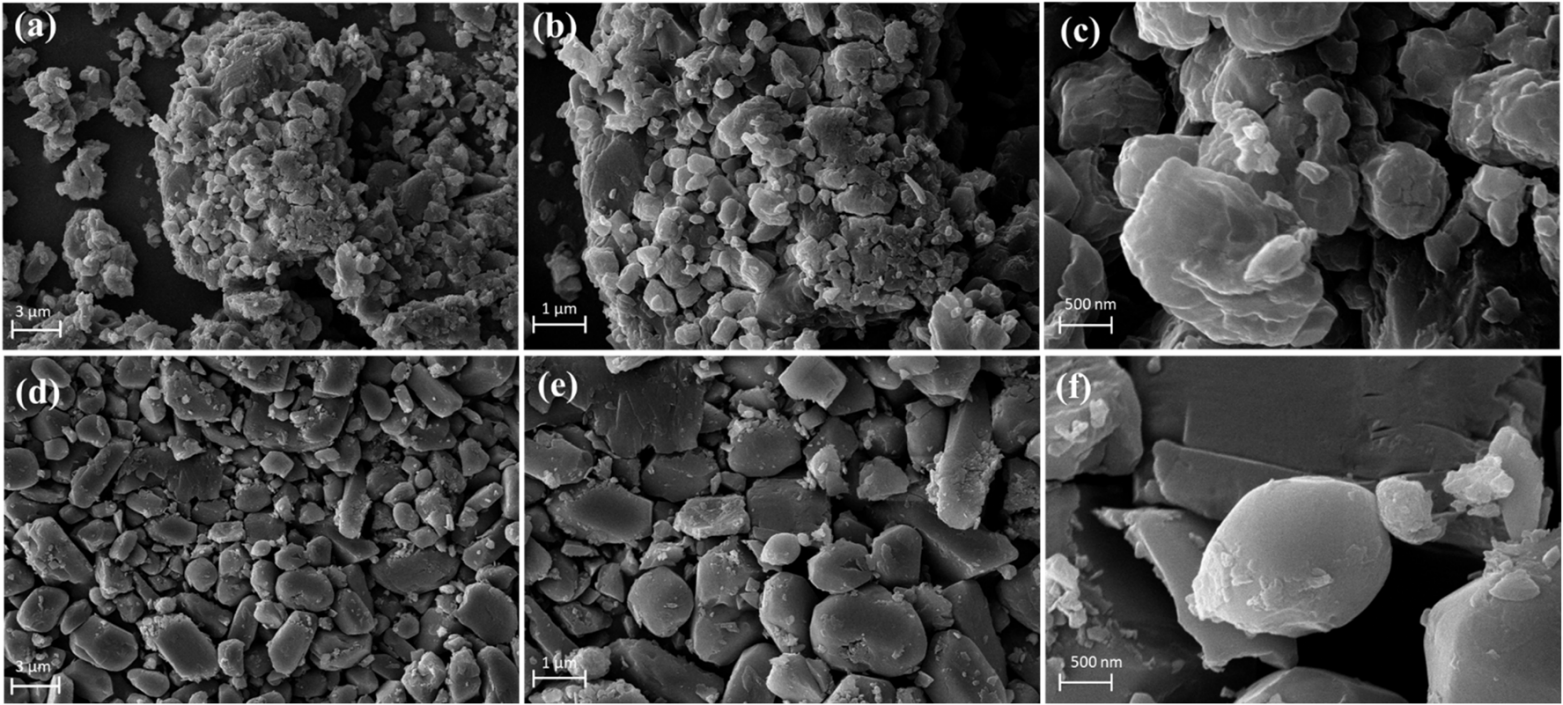
(a–c) SEM images of Co-MOF, synthesized *via* 5-nitroisophthalic acid (X1), (d–f) SEM images of Co-MOF, synthesized *via* pyridine-2,6-dicarboxylic acid (X2) at different resolution.

The Fig. 2 illustrate the SEM images and morphological differences between two cobalt-based metal–organic frameworks (Co-MOFs), both designed as electrocatalysts for water splitting. The Fig. 2(a–c) represents the Co-MOF synthesized using 5-nitroisophthalic acid as the linker, while Fig. 2(d–f) showcases the Co-MOF synthesized with pyridine-2,6-dicarboxylic acid as the linker at different resolution. Both materials exhibit promising features for electrocatalytic applications, with the latter demonstrating superior structural attributes that likely enhance its overall catalytic performance. The X1 exhibits a dense, aggregated microstructure with smaller and irregularly shaped flakes. These clusters provide a high surface area, which is beneficial for catalytic reactions as it increases the number of available active sites. The material's morphology facilitates adequate electrolyte infiltration and ion transport, enabling it to achieve notable performance in both the oxygen evolution reaction (OER) and hydrogen evolution reaction (HER).^43^ However, the lack of uniformity and interconnected pores introduces diffusion limitations, potentially restricting the accessibility of deeper catalytic sites. Moreover, the densely packed structure may lead to localized resistance, which can reduce the overall reaction kinetics.^44^ Despite these limitations, this Co-MOF exhibits significant catalytic activity, showcasing its viability as an electrocatalyst for water splitting. In contrast, the X2 demonstrates a more organized plate-like morphology with well-defined, larger flakes and a layered structure. This uniform and structured morphology enhances electrolyte infiltration and ion accessibility, ensuring efficient interaction between the catalyst and reactants.^44^ The flat, interconnected flakes expose a higher proportion of active sites, which is crucial for accelerating reaction kinetics in both HER and OER. Additionally, the improved pore distribution and structural integrity reduce diffusion barriers, promoting better mass and charge transport.^45^ These morphological attributes are also expected to exhibit superior long-term stability, as its pyridine-based framework provides stronger coordination bonds that resist hydrolysis and structural degradation under harsh electrochemical conditions.^46^ Moreover, the intrinsic electronic properties of the pyridine-2,6-dicarboxylic acid linker could further improve reaction kinetics, particularly for the OER, where efficient O–O bond formation is critical. These attributes make it an excellent candidate for sustainable and practical water-splitting applications.

**Fig. 3:**
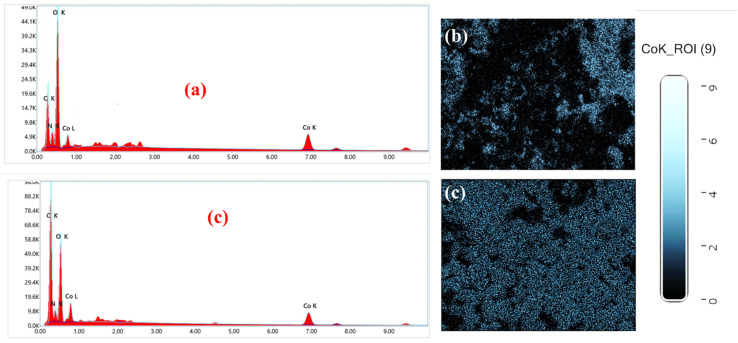
(a) EDX results for elemental analysis of X1, (b) Co distribution map of X1, (c) EDX results for elemental analysis of X2, (b) Co distribution map of X2.

The obtained MOFs nanomaterials were characterized with the help of energy-dispersive X-ray spectroscopy (EDX) in order to uncover their elemental compositions. Fig. 3(a) displays the EDX spectra obtained for X1 and X2 correspondingly. The EDX spectra of X1 reveal prominent peaks corresponding to cobalt, carbon (C), oxygen (O), and nitrogen (N). The distribution maps (Fig. 3(b) and S3†) show a relatively uniform dispersion of cobalt throughout the material, ensuring a high density of catalytic sites. However, slight variations in elemental distribution are observed, potentially arising from the aggregated morphology seen in the SEM images. This aggregation could result in localized regions of varying activity, which might impact overall catalytic performance.^47^ Nevertheless, the elemental composition aligns well with the expected stoichiometry, affirming the successful synthesis of the material (Fig. 3(c)). In the case of X2, the EDX analysis similarly identifies cobalt, carbon, oxygen, and nitrogen as the primary elements. The nitrogen signal, originating from the pyridine ring in the linker, indicates strong coordination with the cobalt centers. The elemental maps (Fig. 3(d) and S4†) show an exceptionally homogeneous distribution of cobalt and nitrogen, reflecting the well-defined and layered morphology observed in the SEM images. This uniformity enhances the accessibility of active sites, contributing to the superior catalytic performance of this MOF.^18^ The well-dispersed cobalt atoms are indicative of a stable coordination environment, which is critical for maintaining structural integrity during electrochemical operations.

### 3.2. Electrochemical study

**Fig. 4:**
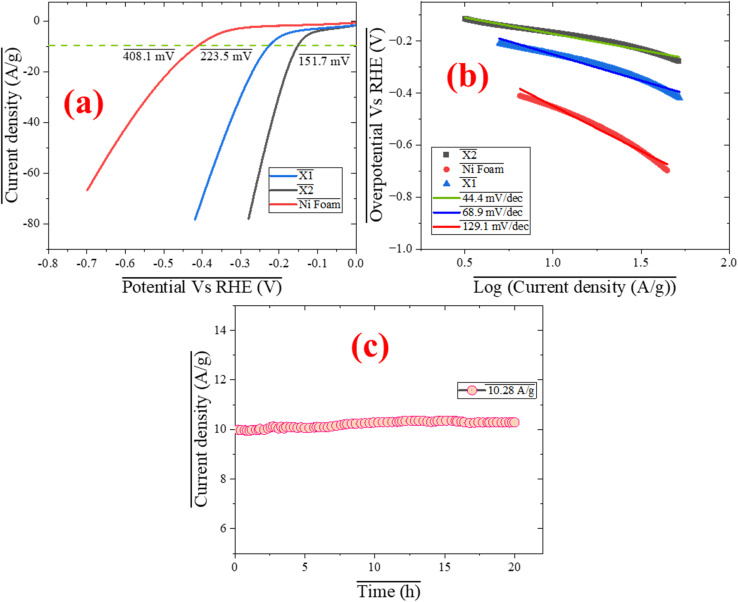
(a) HER polarization curves of X1, X2 and bare Ni foam (b) Taffel slope, (c) stability test outcome of X2 while being operated at 10 A g^−1^.

The Fig. 4 provides a comprehensive evaluation of the electrocatalytic performance of two Co-MOFs synthesized with different linkers, X1 (with 5-nitroisophthalic acid) and X2 (with pyridine-2,6-dicarboxylic acid), for the HER under alkaline condition of 1 M KOH. Fig. 4(a) demonstrates HER polarization curves, relationship between the applied voltage (overpotential) and the resulting current density during the hydrogen evolution reaction (HER) in an electrochemical system. X2 exhibits the lowest overpotential of 151.7 mV at 10 A g^−1^, demonstrating superior catalytic activity compared to X1 (223.5 mV) and Ni foam (408.1 mV). This enhanced performance of X2 can be attributed to its uniform morphology and better elemental dispersion, as evidenced by SEM and EDX analyses, which reveal well-dispersed cobalt active sites that facilitate more efficient hydrogen evolution. X1, while less efficient than X2, still outperforms bare Ni foam, suggesting that the Co-MOF structure contributes to improved HER performance by increasing the number of active sites available for the reaction. Fig. 4(b) displays the Tafel slopes derived from the HER polarization curves, which provide critical insights into the reaction kinetics and efficiency of the catalysts. In this case, X2 exhibits a Tafel slope of 44.4 mV dec^−1^, indicating relatively fast reaction kinetics.^48^ This suggests that the catalyst facilitates the HER with greater efficiency. The lower Tafel slope is likely due to the well-dispersed cobalt active sites in X2, which enhance the adsorption and desorption of protons, key steps in the HER mechanism.^49^ Additionally, the pyridine-2,6-dicarboxylic acid linker in X2 likely contributes to the efficient electron transfer between the active sites and the electrolyte, further accelerating the reaction. Additionally, this Tafel slope suggests that the rate-determining step of the HER mechanism on X2 may involve proton adsorption (the Volmer step) or a combination of the Volmer and Heyrovsky steps, both of which are efficiently facilitated by the material's structure. X1 has a higher Tafel slope of 68.9 mV dec^−1^, indicating relatively slower reaction kinetics. Bare Ni foam has the highest Tafel slope of 129.1 mV dec^−1^, confirming its poor HER kinetics. Ni foam likely relies on the Tafel step (the recombination of two hydrogen atoms to form H_2_), which is less efficient in the absence of a suitable catalyst, thus requiring a higher overpotential. In Fig. 4(c) the long-term stability of X2 is assessed by applying a constant potential of 151.7 mV (its overpotential at 10 A g^−1^) for 20 hours. The current density remains stable throughout this period, with only slight variation increased to 10.28 A g^−1^, demonstrating the robust structural stability and excellent durability of X2. The minimal variation in current density during the 20 hours stability test highlights X2's durability, demonstrating its ability to maintain performance and active sites, making it a promising candidate for practical HER applications.

**Fig. 5:**
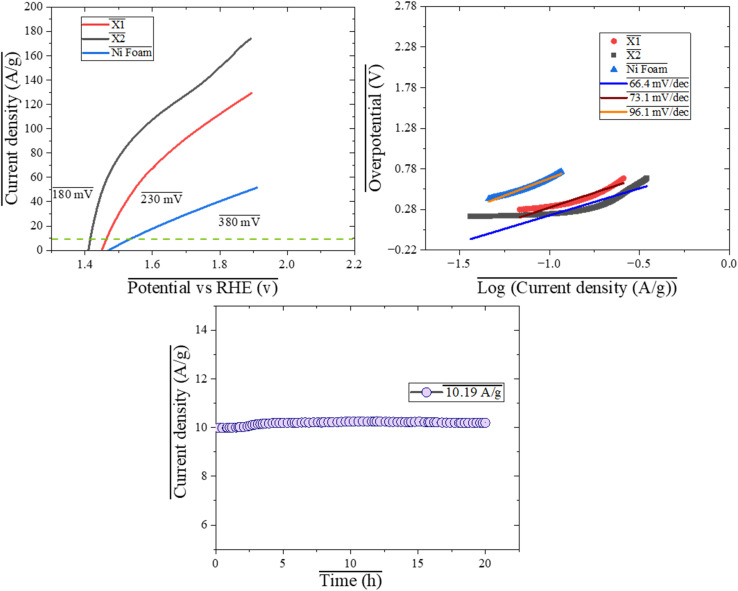
(a) OER polarization curves of X1, X2 and bare Ni foam (b) Taffel slope, (c) stability test outcome of X2 while being operated at 10 A g^−1^.

The electrocatalytic performance of X1, X2, and Ni foam for the oxygen evolution reaction (OER) was evaluated through polarization curves, Tafel slopes, and stability tests. The OER polarization curves shown in Fig. 5(a) illustrate the overpotential required to achieve the corresponding current densities. To achieve 10 A g^−1^ X1, the required overpotential was 230 mV, while X2 exhibited a lower overpotential of 180 mV. Ni foam, required a higher overpotential of 380 mV. These results indicate that X2 outperforms both X1 and Ni foam in terms of electrocatalytic efficiency. A lower overpotential implies that X2 is able to drive the OER with less energy input, which is an important feature for enhancing the energy efficiency of electrochemical systems. The reduced overpotential of X2 can be attributed to its superior catalytic properties, which allow it to facilitate the OER more effectively than the other catalysts.^50,51^ To gain further insight into the reaction kinetics of the catalysts, the Tafel slopes derived from the OER polarization curves are shown in Fig. 5(b). The Tafel slopes for X1, X2, and Ni foam were 73.1 mV dec^−1^, 66.4 mV dec^−1^, and 96.1 mV dec^−1^, respectively. A lower Tafel slope again suggests faster reaction kinetics, indicating that the catalyst promotes the OER at a faster rate. X2 exhibited the lowest Tafel slope of 66.4 mV dec^−1^, demonstrating that it facilitates the OER with the most efficient kinetics among the three catalysts. In comparison, X1 (73.1 mV dec^−1^) and Ni foam (96.1 mV dec^−1^) displayed slower kinetics, with Ni foam showing the least efficient performance in terms of reaction rate. These observations suggest that X2 not only requires less overpotential to achieve the same current density but also promotes the reaction more rapidly, making it a highly effective catalyst for OER. The long-term stability of X2 was assessed through a stability test, where a constant potential of 180 mV (the overpotential of X2) was applied for 20 hours (Fig. 5(c)). The current density at the start of the experiment was 10 A g^−1^, and after 20 hours, it increased only slightly to 10.19 A g^−1^. This variation in current density indicates the excellent stability and durability of X2 under prolonged electrochemical conditions. The near-constant current density over the 20 hours period demonstrates that X2 retains its catalytic performance without significant degradation, underscoring its robust structural integrity and sustained electrocatalytic activity. The small increase in current density can be attributed to slight variations in the electrochemical environment or the accumulation of reaction intermediates, but the fact that the change is negligible reinforces the stability of X2.

**Fig. 6:**
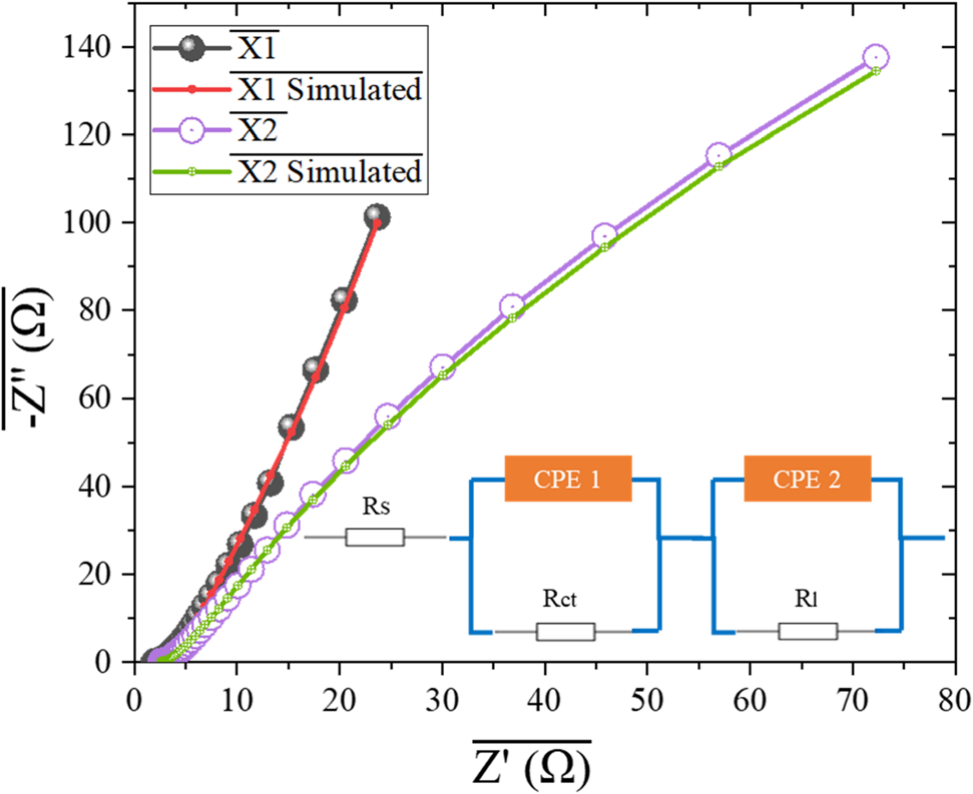
Electrochemical impedance spectroscopy of both synthesized MOFs with inset showing high frequency trend of spectrum.

The Fig. 6 presents the Electrochemical Impedance Spectroscopy (EIS) measurements for two materials, X1 and X2, which are studied in the context of their OER and HER. The Nyquist illustrates the EIS data for X1 and X2, along with their corresponding fitted curves. To analyze the charge transfer properties of these catalysts, an equivalent circuit model was employed, as shown in the inset, consisting equivalent series resistance (*R*_s_), charge transfer resistance (*R*_ct_), load resistance (*R*_l_) and two constant phase elements (CPE1 and CPE2) representing the non-ideal capacitive behavior at the electrode–electrolyte interface. Fitted impedance values are presented in Table 1. For X1 the Nyquist plot reveals a larger semicircular arc, indicating a higher charge transfer resistance and slower electron transfer kinetics. This correlates with the material's relatively poor electrochemical performance observed in both OER and HER, which can be attributed to its irregular morphology and structural limitations. The high resistance in the Nyquist plot is a direct consequence of inefficient charge transfer processes, which hinder the material's overall catalytic activity. In contrast, X2 exhibits a smaller semicircular arc in the Nyquist plot, and a lower charge transfer resistance and more efficient electron transfer. The EIS data for X2 indicates improved electrochemical behavior, which aligns with its superior catalytic activity in both OER and HER.^52^ This enhanced performance is supported by the SEM and EDX results, which show a more uniform morphology and better dispersion of active sites, promoting faster reaction kinetics. These findings underline the importance of material design in optimizing the electrocatalytic properties. The optimized performance of X2, characterized by its high stability and enhanced reaction kinetics, underscores its promise as a sustainable solution for green energy technologies compared to recently reported literature.^53–57^ Such advancements bring us closer to realizing the vision of efficient and scalable renewable energy systems for a more sustainable future.

**Table 1:** EIS fitting outcomes

Parameter	X1 (Ω)	X2 (Ω)
*R*_s_	0.86	0.65
*R*_ct_	1.8	0.89
CPE1	0.85 mF	1.2 mF
CPE2	0.67 mF	1.05 mF

## 4. Conclusion

This study systematically explores how variations in linker chemistry affect metal-active site distribution, stability, and reaction kinetics, providing a deeper understanding of structure/morphological-performance relationships. The synthesis and characterization of two Co-MOFs, synthesized with distinct linkers, 5-nitroisophthalic acid (X1) and pyridine-2,6-dicarboxylic acid (X2), was achieved *via* hydrothermal approach. X1 exhibits suboptimal catalytic performance, whereas X2 demonstrates superior structural integrity, and uniform morphology. The X2 demonstrated outstanding catalytic performance with a low overpotential of 151.7 mV and a Tafel slope of 44.4 mV dec^−1^ for the hydrogen evolution reaction (HER), while for the oxygen evolution reaction (OER), X2 required a lower overpotential of 180 mV and exhibited a Tafel slope of 66.4 mV dec^−1^, outperforming X1 and bare Ni foam. It is also coupled with remarkable stability over 20 hours continuous operation. By linking catalytic efficiency to the structural and morphological effects govern by linker selection, this work offers new insights that can guide the rational design of future MOF-based electrocatalysts, paving the way for further advancements in sustainable hydrogen production.

## Data availability

The data will be made available on request.

## Conflicts of interest

There are no conflicts to declare.

## Supplementary Material

RA-015-D5RA00286A-s001
