# Supplementary material for: From design to efficiency: cobalt-based MOFs for efficient and stable electrocatalysis in hydrogen and oxygen evolution reactions
Source: RSC Adv. 2025 Mar 18;15(11):8420–9. doi: 10.1039/d5ra00286a (PMC11917210; doi:10.1039/d5ra00286a)
Supplement: RA-015-D5RA00286A-s001 [file RA-015-D5RA00286A-s001.pdf]

Supplementary Information (SI) for RSC Advances.
This journal is © The Royal Society of Chemistry 2025

From Design to Efficiency: Cobalt-Based MOFs For Efficient and Stable Electrocatalysis in Hydrogen and Oxygen Evolution Reactions

Junaid Khan[a, b, *], Anique Ahmed[c, d], Abdullah A. Al-Kahtani[e]

[a]Department of Physics, Government Postgraduate Collage No.1, Abbottabad, Khyber Pakhtunkhwa, Pakistan

[b]Department Of Higher Education Achieves and Libraries, Government of Khyber Pakhtunkhwa, Pakistan

[c]Faculty of Engineering Sciences, GIK Institute of Engineering Sciences and Technology, Topi 23640, Khyber Pakhtunkhwa, Pakistan

[d]Department of Chemical and Bilogical Engineering, Gachon University, 1342 Seongnam-daero, Seongnam13120, Republic of Korea

[e]Chemistry Department, Collage of Science, King Saud University, P. O. Box 2455, Riyadh-22451, Saudi Arabia

*Email: junaidkhan.nanotech@gmail.com

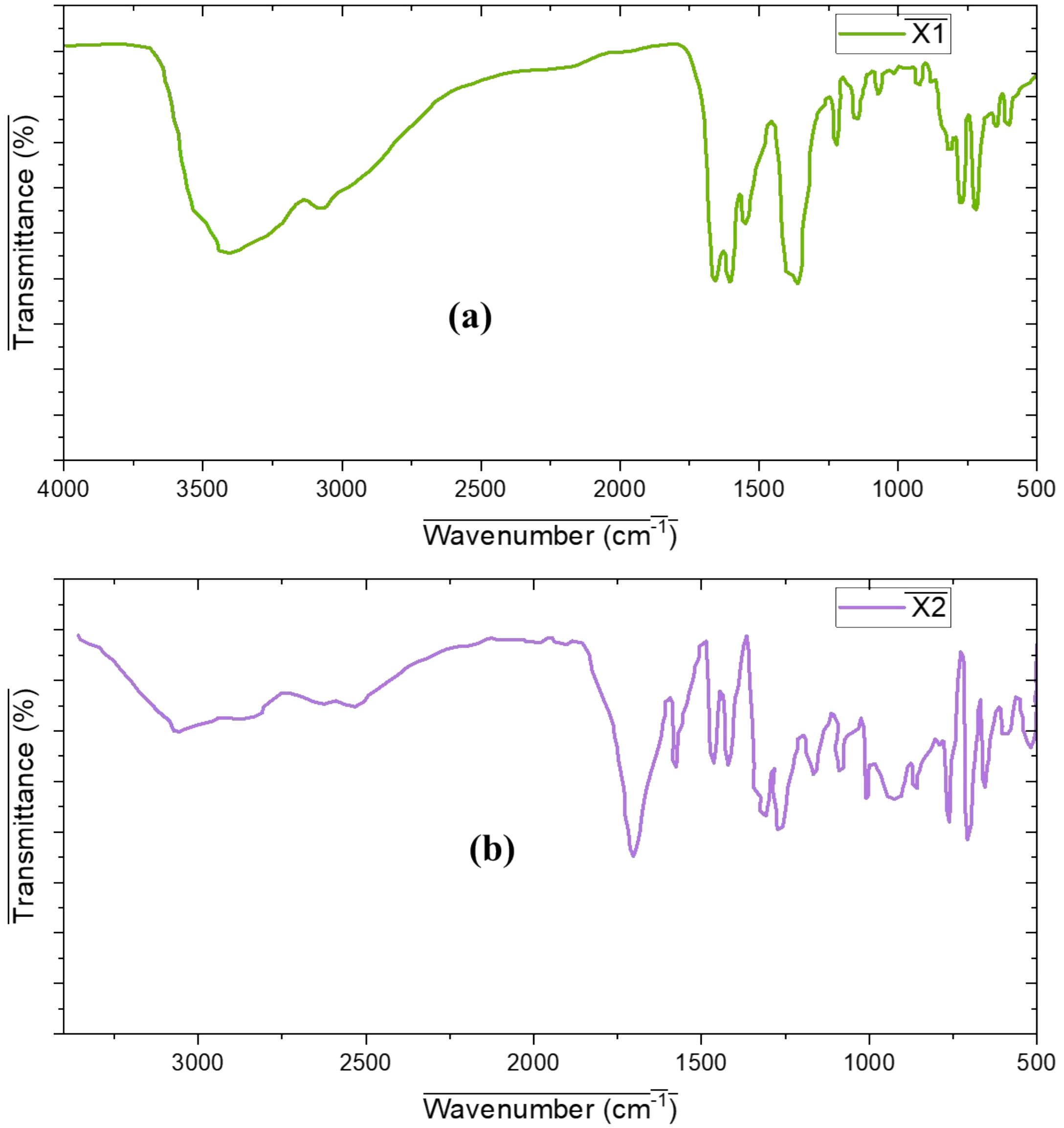

Figure S1: FTIR spectrum of X1 and X2.

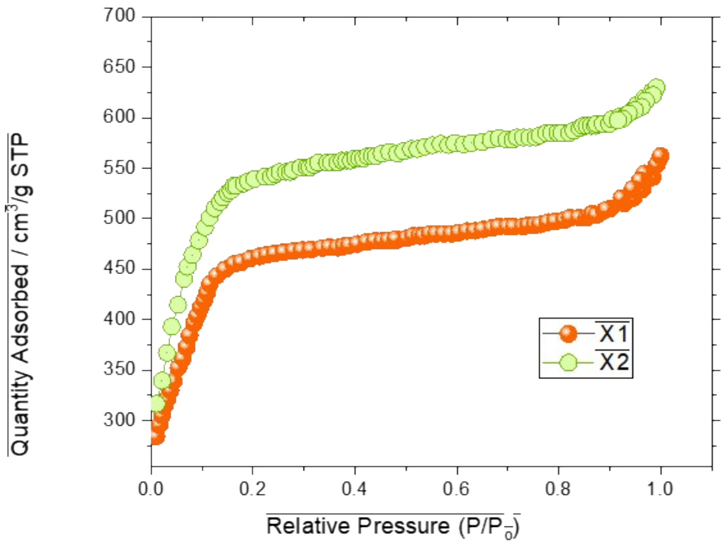

Figure S2: BET results of X1 and X2

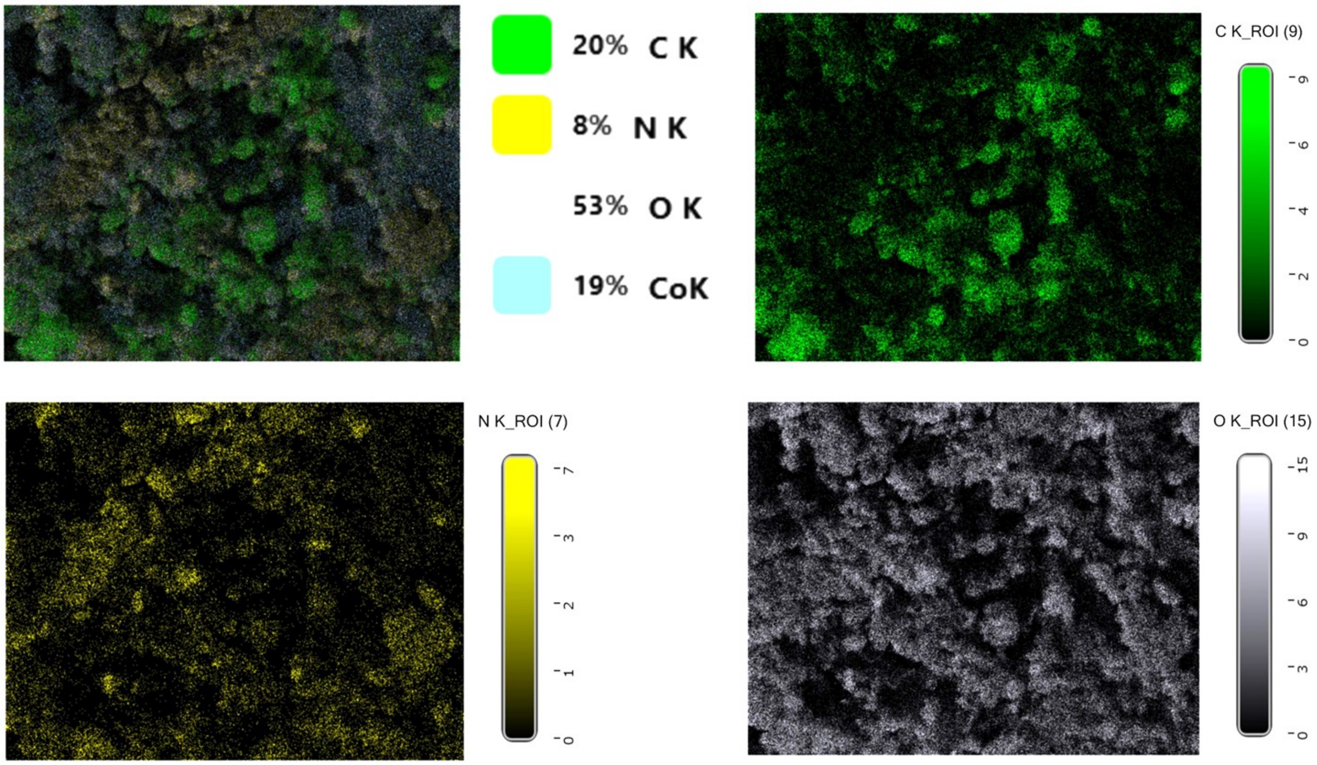

Figure S3: Elemental mapping of X1.

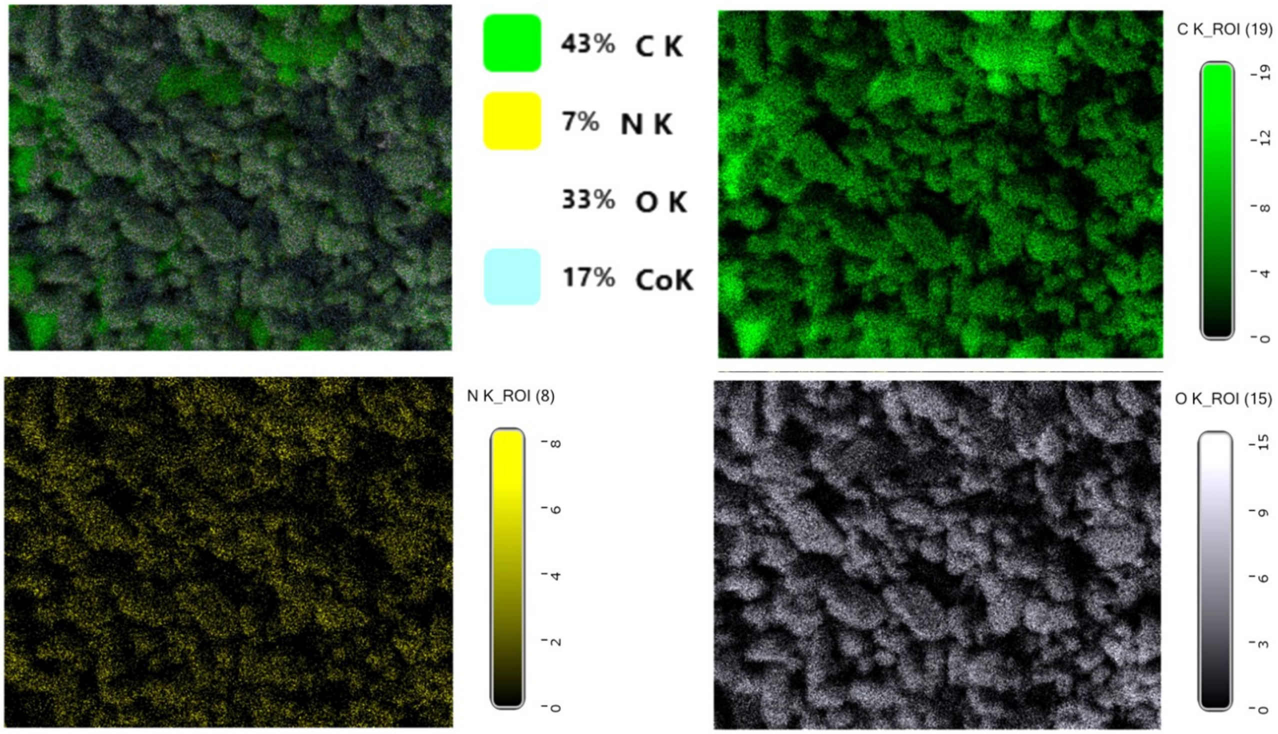

Figure S4: Elemental mapping of X2.
